# A rapid review of guidelines on the involvement of adolescents in health research

**DOI:** 10.1111/hex.14058

**Published:** 2024-06-10

**Authors:** Azza Warraitch, Ciara Wacker, Delali Bruce, Ashling Bourke, Kristin Hadfield

**Affiliations:** ^1^ Trinity Centre for Global Health, Trinity College Dublin Dublin Ireland; ^2^ School of Psychology, Trinity College Dublin Dublin Ireland; ^3^ School of Engineering Stanford University Stanford California USA; ^4^ Institute of Education Dublin City University Dublin Ireland

**Keywords:** adolescent engagement, adolescent involvement, guidelines, public and patient involvement

## Abstract

**Background:**

Meaningful involvement of adolescents in health research is their fundamental human right and has many benefits. A lack of awareness among researchers on how to meaningfully involve adolescents in health research has been linked to adolescent under involvement in health research. To address this barrier, studies have reported the need for more guidance. To inform the development of better guidelines on adolescent involvement, there is a need to first consolidate the currently available guidance on adolescent involvement in health research and to identify the gaps in these guidelines. This review aims to systematically identify all the currently available guidelines on adolescent involvement in health research and evaluate their scope, content, context, and quality.

**Methods:**

This rapid review was pre‐registered with PROSPERO #CRD42021293586. It included documents that incorporated tangible recommendations on the involvement of adolescents in health research. We searched six databases for peer‐reviewed literature: MEDLINE, CINAHL, Embase, Scopus, Web of Science, and ERIC. We conducted a grey literature search in Google Scholar, Google, websites of 472 relevant organisations and sought expert input. The quality of the guidelines was assessed using the Appraisal of Guidelines for REsearch & Evaluation (AGREE‐II) Instrument. Data was analysed using descriptive analyses and narrative synthesis.

**Results:**

We found that the current guidelines on adolescent involvement in health research are often narrow in scope, targeting specific users and populations while focusing on limited research areas. The guidelines individually fail to provide comprehensive coverage of recommendations across all topics related to adolescent research involvement, that are collectively addressed across all included guidelines. Furthermore, these guidelines tend to be context‐specific and are generally of low quality, often due to inadequate stakeholder involvement and a lack of rigorous development methods.

**Conclusion:**

This review provides a consolidated list of guidelines on adolescent involvement in health research along with their quality scores as a resource for researchers to select the guidelines suitable for their research topic, context, and scope for adolescent involvement. There is a need to develop a set of guidelines on adolescent involvement in research, which are comprehensive in scope, cover all key aspects of adolescent involvement in health research, can be adapted for different contexts, and which are based on rigorous and systematic methods.

**Patient and Public Involvement:**

Adolescent co‐researchers D. B. and C. W. were involved at different stages of the review process. D. B. screened 25% of the peer‐reviewed articles at the title and abstract screening stage and 10% at full‐text screening stage. C. W. extracted data from 10% of the included guidelines. Both co‐researchers reviewed and shared their feedback on the article and are co‐authors on this paper. They will also be invited to contribute to further dissemination of the findings from this review.

## 1 INTRODUCTION

Twenty‐four percent of the world's population is comprised of adolescents aged 10–24 years.^1^ There has been a growing focus on research on adolescent health in the past two decades,^2^, ^3^ accompanied by an increased recognition of the importance of involving adolescents in research on adolescent health.^3^, ^4^ This is defined as ‘research that is done “with” or “by”’ young people, ‘not “to,” “about” or “for” them’.^5^, ^6^ The emphasis on adolescent involvement in health research in recent years has been linked to several factors. *First*, researchers and communities are increasingly becoming cognisant of adolescents' right to have a say in all decisions that affect them, as outlined in article 12 of the United Nations Convention on the Rights of the Child.^7^ *Second*, there is growing evidence on the benefits of adolescent involvement in health research for adolescents, research, and the researchers.^6^, ^8^, ^9^ *Third*, there is an increased awareness of the sociological theories that recognise adolescents as independent actors with agency.^10^ *Fourth*, adolescent involvement is perceived as a way to co‐create knowledge with the target population^11^ and to promote transparency and accountability in research practices.^10^ This, in turn, can result in research that is more rigorous and credible, and better aligned with adolescents’ needs.^10^ These factors have contributed to a heightened demand for public and patient involvement of adolescents in health research from organisations and funding bodies.^3^, ^12^, ^13^, ^14^

Despite the widespread recognition of the importance of their involvement, adolescents remain underinvolved in health research.^6^ A recent review by Sellars et al. found that less than 1% of studies on child and adolescent health research involved adolescents as advisors.^4^ In contrast to adults' involvement in health research, adolescents are less often involved.^15^ This is attributed to multiple barriers experienced by researchers at the researcher, adolescent, and organisational levels.^4^ One of the most critical researchers‐level barriers to adolescent involvement in health research is a lack of awareness among researchers on different aspects of adolescent involvement.^6^ This includes a lack of awareness of the significance and benefits of adolescent involvement in health research, a limited understanding of how to involve adolescents meaningfully and ethically, and misconceptions about adolescents’ skills to contribute to health research.^6^, ^16^, ^17^ In a study by Hawke et al., 45% of interviewed researchers reported a lack of knowledge of how to engage young people practically.^18^ Among these, 49% admitted to not involving young people in their research due to a lack of understanding of youth involvement. Moreover, 69% of researchers highlighted the need for additional training for researchers on engaging young people in health research, while 42% expressed the demand for more enhanced curriculum and resources on youth involvement.^18^ Similarly, in Das et al.' stakeholder consultations with researchers and adolescents, two‐thirds of researchers expressed a need for more guidance materials on young people's involvement in health research. Researchers' rationale for this observation was rooted in the fact that much of their knowledge in this field is acquired through on‐the‐job learning and guidance from supervisors.^19^ The call for more training and comprehensive resources for researchers on involving adolescents in health research has been echoed in multiple studies.^6^, ^15^, ^16^, ^20^, ^21^

To inform the development of further guidelines on adolescent involvement in health research, there is a need to first consolidate the currently available resources in this area and to identify the gaps in the current guidance that need to be addressed in subsequent guidelines. Four studies have reviewed some of the available guidance on adolescent involvement in health research,^6^, ^22^, ^23^, ^24^ however these studies are limited in several ways. For example, some of these studies focused on providing a brief overview of a few most used frameworks to guide youth participation in research^6^, ^22^, ^23^ or some of the approaches used to engage adolescents in research.^24^ However, these studies exclusively focused on frameworks and approaches to youth involvement in health research and did not review the currently available practical guidance, such as toolkits and manuals on adolescent involvement in health research. Furthermore, Wilson et al. provided a list of 14 guidelines for young people's involvement in health research based on their overview of literature on adolescent involvement in health research and stakeholder consultations with researchers. They found these guidelines to be limited in multiple areas and underscored the need for more comprehensive guidelines to assist researchers in effectively involving adolescents in health research.^6^, ^19^ However, while Wilson et al. provided a comprehensive overview of the literature on overall adolescent involvement in health research, their review did not aim to identify all the currently available guidance on this topic. In contrast to their review, the Patient Experience Library initiative consolidated a list of available guidance on overall public and patient involvement in research.^25^ They identified 536 frameworks and guidelines for involving the public in research and 28 guidelines for involving youth in research. However, neither Wilson et al. nor the Patient Experience Library initiative systematically reviewed and assessed all available guidelines on adolescent involvement in health research. However, this is crucial not only to prevent duplicative efforts but also to address the limitations present in current guidelines and build upon their included recommendations. To achieve this, we conducted this rapid review to identify available guidance, including existing frameworks, practical guidelines, toolkits and manuals on adolescent involvement in health research and to evaluate the scope, content, contexts, and quality of these existing guidelines.

## 2 METHODS

This rapid review was conducted as per the Guidance from the Cochrane Rapid Reviews Methods Group.^26^ We followed the Preferred Reporting Items for Systematic Review and Meta‐Analysis (PRISMA) statement^27^ in reporting this review given that the PRISMA‐Rapid Review extension is under development.^28^ The rapid review was pre‐registered with the International Prospective Register of Systematic Reviews (PROSPERO CRD42021293586). All relevant materials—including the search strategy and results—can be accessed on the Open Science Framework (OSF) page for this review: https://osf.io/au6dh/?view_only=621932b8ee274fa5b85876b92cf62e7a.

### 2.1 Search strategy

The search strategy for this rapid review was based on keywords for: (i) target population (adolescents aged 10–24 years),^29^ (ii) exposure (involvement of adolescents in research), (iii) study type (guidelines, manuals, toolkits, frameworks, papers with recommendations) using Boolean syntax (Supporting Information S1: Table 1). A. W. (a PhD student in psychology) developed the search strategy in consultation with K. H. (an assistant professor in psychology at Trinity College Dublin) and a research librarian (G. F.) at Trinity College Dublin.

### 2.2 Information sources

#### 2.2.1 Electronic databases for peer‐reviewed literature

We searched MEDLINE, CINAHL, Embase, Scopus, ERIC and Google Scholar to identify relevant guidelines on adolescent involvement in health research, published before 31 December 2021. No restrictions for year or study design were applied in the search strategy.

#### 2.2.2 Grey literature search

Given that most guidelines are published as grey literature, we conducted a thorough grey literature search. First, we searched the Web of Science. Second, we searched the first 10 pages of Google for relevant guidelines, using a simplified search strategy. Third, we searched websites of organisations that work on adolescent health research. To do this, we first identified the relevant organisations by searching up to 20 pages of Google using keywords related to adolescent involvement in health research, browsed the Mental Health Innovation Network (MHIN) database, and included relevant organisations known to the research team. The search strategy was piloted before pre‐registration, and the decision to limit the Google search to 10 pages for guidelines and 20 pages for youth health organisations was based on the relevance of results found within these page limits. The MHIN database includes a list of organisations working on health promotion research across the globe. Additionally, we searched Google for youth health organisations in each of the 137 low‐ and middle‐income countries. We used the World Bank Country and Lending Groups data from 2021 for the list of all low‐, middle‐, and high‐income countries, resulting in a total of 137 countries. We searched the website of the organisation that was the top Google result for each of these 137 countries. Through these sources, we compiled a list of 472 organisations working on adolescent health research in low‐, middle‐ and high‐income countries. We then used a simplified version of the search strategy to browse the websites of these 472 organisations to identify relevant guidelines and materials. We hand‐searched the websites that did not have a search option. Fourth, we sought input from experts on youth involvement who were either researchers with expertise in this area or were professionals in charge of engaging adolescents at youth health organisations. The experts were from the United Kingdom, United States, and Australia and were asked to share relevant sources and materials on adolescent engagement with which they were familiar.

### 2.3 Eligibility criteria

#### 2.3.1 Study design

We included all guidelines, including frameworks, toolkits, practice documents, manuals, papers, and websites which included a set of tangible recommendations on the involvement of adolescents at any stage of the research process. Guidelines did not have to be based on systematic reviews; this is because there are limited guidelines on public and patient involvement that were developed using systematic methods.

#### 2.3.2 Participants

We were interested in guidelines that were specific to adolescents. Adolescents are aged 10–24 years as defined by Sawyer et al.^29^ However, since most of the included guidelines did not specify the age range of the target population, we included all guidelines that were intended for involvement of children and adolescents in research.

#### 2.3.3 Intervention

We initially aimed to include guidelines that focused on adolescent involvement in health research. However, given that a very limited number of guidelines were focused on adolescent involvement in health research, we included all guidelines that aimed to provide recommendations or instructions for involving adolescents in overall research. We defined adolescent involvement as ‘research that is done “with” or “by”’ young people, ‘not “to”, “about” or “for” them’.^5^, ^6^ Adolescent involvement is a very broad term that is used to refer to a wide range of methods and approaches. Therefore, to ensure inclusion of all the relevant materials, we included guidelines focusing on all types and levels of adolescent involvement.

#### 2.3.4 Outcomes

We included guidelines that incorporated a set of tangible recommendations on involvement of adolescents in health research and provided instructions for how to involve them. We excluded articles and guidelines that only focused on describing how adolescents were involved in a project.

#### 2.3.5 Other criteria

Only guidelines published in the English language were included. Guidelines behind a paywall that the Trinity College Dublin library did not have access to were excluded.

### 2.4 Selection of studies

**Figure 1:**
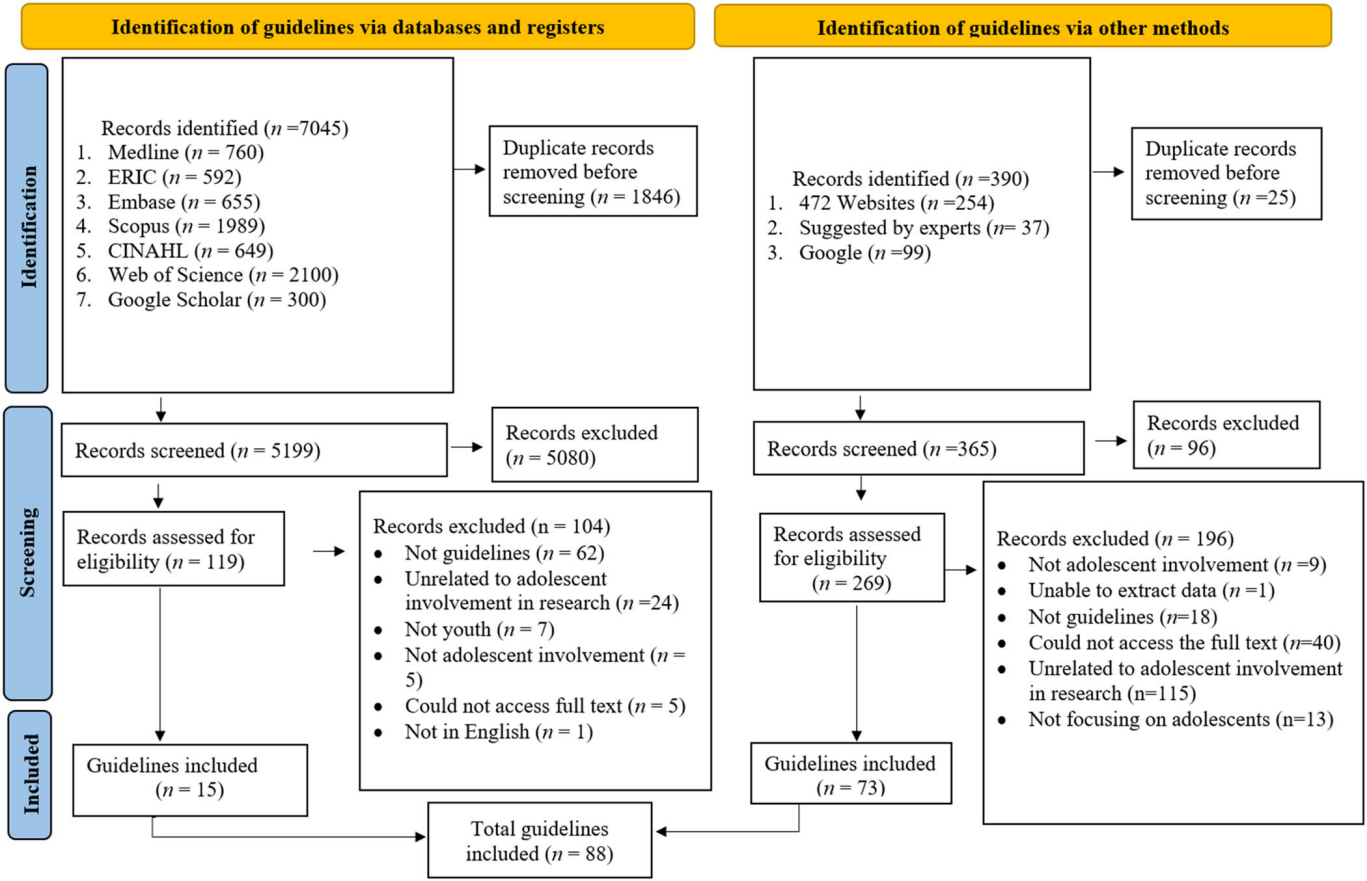
Preferred Reporting Items for Systematic Reviews and Meta‐Analyses flowchart. One of the guidelines was 200 pages long and consisted of screenshots of text rather than text that could be copied and pasted, which hindered our ability to extract data due to logistical constraints.

Search results from electronic databases for peer‐reviewed literature were imported into Covidence. Results from the sources for grey literature were imported into an Excel sheet. Title and abstract screening and full‐text screening was conducted by two researchers. AW conducted the title and abstract screening of all articles, while an adolescent co‐researcher (D. B.) conducted the title and abstract screening of 25% of peer‐reviewed articles to minimise the risk of bias. Similarly, A. W. conducted the full‐text screening and extracted data from all eligible reports, while an adolescent co‐researcher C. W. conducted full‐text screening for 10% of the peer‐reviewed full‐text articles and data extraction from 10% of included guidelines. Any discrepancies or disagreements were resolved through discussion and, if required, through discussion with a third researcher (K. H.). The study selection process is reported in Figure 1.

### 2.5 Data extraction

Data was extracted on the characteristics of the included guidelines (e.g., target population, target users, health research areas, guideline development methodology, organisations involved, etc.) and recommendations on involving adolescents in health research (e.g., preparing for involving adolescents in health research, identifying and recruiting adolescents, and addressing barriers to adolescent involvement, etc.) into an Excel sheet.

### 2.6 Quality assessment

We used the Appraisal of Guidelines for REsearch & Evaluation (AGREE) II Instrument^30^ to assess the quality of the included guidelines. We used this instrument because it accounts for the wide variability in the guideline development methods and gives greater weight to stakeholder involvement in the guideline development process. This tool assesses the quality of guidelines across six domains including clarity of the scope and purpose of the guidelines, stakeholder involvement in the development process, rigour of development, applicability of guidelines, clarity of presentation, and editorial independence. While the items are rated on a 7‐point Likert scale in the original instrument, we adapted it to simplify to a three‐point Likert scale with 1 = Strongly Disagree, 2 = Somewhat Agree and 3 = Strongly Agree. We modified the original 7‐point Likert scale to a simpler 3‐point Likert scale to better match the quality assessment process for the included guidelines. When rating, we focused on whether certain indicators were present or absent within the guidelines. This streamlined the assessment by offering options for somewhat or fully agreeing. For instance, when considering the involvement of adolescents or the target population, we rated as strongly disagree if absent, somewhat agree if they were involved to some extent and strongly agree if their involvement was extensive. The scores for all items in each domain were summed and the scaled percentage scores for each domain were calculated using the following formula:

$$\frac{\mathrm{Obtained}\;\mathrm{score}-\mathrm{Minimum}\;\mathrm{possible}\;\mathrm{score}}{\mathrm{Maximum}\;\mathrm{possible}\;\mathrm{score}-\mathrm{Minimum}\;\mathrm{possible}\;\mathrm{score}}\times 100.$$

A. W. conducted the quality assessment for all the included guidelines. The results of the quality assessment are in Table 5 and are also available on the OSF: https://osf.io/au6dh/?view_only=621932b8ee274fa5b85876b92cf62e7a.

### 2.7 Analyses

We used descriptive analysis and narrative synthesis to analyse the data. Data on year of publication, age range of target population, terminology and adolescent involvement approaches focused on in the guidelines, areas of health research, regions where the guidelines were published, contexts in which the guidelines are applicable, target users of the guidelines, organisations involved in development process, contact details of authors and editorial independence, content covered in the guidelines, and quality assessment scores were quantitatively analysed using descriptive statistics in SPSS v27. Data on the methodology of guideline development and on adolescents' involvement in the guideline development process were narratively synthesised. This involved familiarisation with the extracted data followed by deductive coding of the data. Similar codes were then merged under subthemes and broader themes and were then narratively described.

### 2.8 Patient and public involvement

Adolescent co‐researchers D. B. (high school student, aged 18 years, United States) and C. W. (Undergraduate student, aged 21 years, Ireland) were involved at different stages of the review process. D. B. screened 25% of the peer‐reviewed articles at the title and abstract screening stage and 10% at full‐text screening stage. C. W. extracted data from 10% of the included guidelines. Both co‐researchers reviewed and shared their feedback on the article and are co‐authors on this paper. They will also be invited to contribute to further dissemination of the findings from this review.

## 3 RESULTS

We included a total of 88 guidelines (Supporting Information S1: Table 2) from an initial 7435 reports resulting from all sources (Figure 1).

### 3.1 Guideline development

44% of the guidelines were published between 2016 and 2021 (Supporting Information S1: Figure 1). Seventeen percent of the guidelines did not report the publication year. The guideline development methodologies generally comprised some combination of five key components: (1) building on existing resources, (2) conducting a literature review, (3) involving stakeholders, (4) piloting the guidelines, and (5) obtaining reviews and endorsements (Table 1).

**Table 1:** Guideline development methodology.

Materials	Number of guidelines and description of source materials
1. Building on existing guidelines and other sources	
Previous research projects and guidelines	10 guidelines were built on existing guidelines. 18 guidelines were derived from previous research projects. 4 guidelines were based on researchers' personal experiences.
2. Conducting a literature review	
Reviews	20 of the included guidelines were based on different types of literature reviews including: Desk review (*n* = 1) Targeted literature review of documents from United Nations and NGOs (*n* = 1) Review of the international policy and models on adolescent involvement (*n* = 1) Narrative review (*n* = 1) Systematic review (*n* = 1) Rapid review (*n* = 2) Overview of literature (*n* = 4) Literature reviews (*n* = 10)
3. Involving stakeholders in guideline development	
Stakeholder involvement	34 of the included guidelines involved stakeholders other than adolescents in the development process. These stakeholders included community members, experts on adolescent involvement, staff members of organisations, researchers, and representatives from organisations. Fifty‐one of the guidelines included adolescents in the guideline development process.
Methodology of stakeholder involvement	Stakeholders other than adolescents were involved using the following approaches: Conducting a needs assessment Holding workshops to decide the content of the guidelines Reviewing the first draft of the guidelines Discussions and voting in meetings Brainstorming to develop the key components of adolescent engagement Consensus building in a consultation meeting Conducting interviews Online consultations Innovation days
4. Piloting the guidelines	
Content piloted	Six guidelines reported piloting the content with adolescents who shared feedback to improve the guidelines. They piloted the training methods described in the guidelines, activities for adolescent involvement, youth advisory group tool, and the overall guidelines.
5. Review and endorsement of guidelines	
Two of the guidelines were externally reviewed by a wider group of researchers One of the guidelines was endorsed by a steering committee	

#### 3.1.1 Adolescent involvement in the guideline development process

Fifty‐one of the 88 guidelines involved adolescents in their development. Of these 51 guidelines, only 36 provided details on the methodology of adolescent involvement in the guideline development process. The methods of this involvement can be categorised into three key approaches:

*Consultations with adolescents*: Thirty‐one guidelines reported engaging adolescents through consultations. This involved discussions with youth advisory groups,^31^, ^32^, ^33^, ^34^, ^35^ meetings with adolescents,^36^, ^37^, ^38^, ^39^, ^40^ workshops,^41^, ^42^, ^43^, ^44^, ^45^ interviews,^46^ focus group discussions,^22^ webinars to discuss the key content for guidelines,^47^ and conducting listening sessions with adolescents.^23^ Their feedback was sought on various areas, such as training methods,^48^, ^49^ activities,^50^ overall content,^47^, ^51^, ^52^, ^53^, ^54^ and the initial draft of the guidelines.^42^ Adolescents provided input during the piloting phase^39^, ^55^, ^56^, ^57^ and reviewed the final version to enhance relevance.^58^ One guideline documented consultations with adolescents involved for more than a decade in four projects.^59^

*Collaborating with adolescents in guideline development*: Two guidelines involved adolescents as collaborators. One of the guidelines engaged adolescents as interviewers during the guideline development process.^60^ The other was developed based on discussions and voting with an adolescent advisory group to identify what they believed were the most important things for researchers to know.^61^

*Adolescent‐led guideline development*: Three guidelines were developed by adolescents. Adolescents led the write‐up of two sets of guidelines^62^, ^63^ and led the development process of another.^64^ This latter guideline development process involved over 60 meetings to discuss key terminology and content related to adolescent engagement. The meetings were planned by adolescents and involved participatory methods.^64^

*Stages of adolescent involvement*: Among the 51 guidelines detailing adolescent involvement in the development process, only 11 specified the stage at which adolescents were engaged. Notably, seven guidelines involved adolescents at later stages for their review and feedback on the developed guidelines.^39^, ^42^, ^51^, ^55^, ^56^, ^57^, ^58^ Only four guidelines included adolescents at all key stages of the development process, indicating sustained involvement throughout.^61^, ^62^, ^63^, ^64^

### 3.2 Scope of the guidelines

Sixty‐five percent of the guidelines did not specify the age range encompassed within the target population of children and adolescents. Among the guidelines that did provide an age range, 11% (*n* = 10) were focused on adolescents aged 10–24 years. Overall, the guidelines were designed for a diverse audience (Table 2), encompassing funding bodies (*n* = 6); various types of organisations (*n* = 19); researchers, staff members, and other professionals working with adolescents (*n* = 68); community stakeholders (*n* = 12), and adolescents (*n* = 12). Terminology used for describing adolescents' involvement in health research varied across the guidelines, encompassing 23 different terms (Table 3). The predominant terminologies were ‘participation’ (23%), followed by ‘involvement’ (22%) and ‘engagement’ (20%). There was little overlap in different approaches to adolescent involvement across all included guidelines, as most guidelines focused on specific approaches (e.g., peer education).

**Table 2:** Intended target users of the guidelines.

Intended target users		Number of guidelines
Funding bodies	Funders	2
	Commissioners of research working within the National Health Service, Social Care, and Public Health	4
Organisations	Organisations working with adolescents	8
	Humanitarian organisations	3
	All organisations	3
	Youth participation organisations	2
	Organisations working on peer education	1
	Strategic bodies that plan, commission, and deliver services used by children and adolescents	1
	Research agencies and those developing products or resources for children and adolescents	1
Individuals working with adolescents	Researchers	22
	Staff	9
	Professionals working with adolescents	8
	Policymakers and decision makers	6
	Programme managers	4
	Trainers	4
	Individuals working on peer education	4
	Directors	2
	Anyone interested in involving adolescents in research	2
	Other relevant professionals	2
	Programme coordinators	1
	Supervisors	1
	Monitoring and evaluation officers	1
	Digital health intervention designers, developers, implementers	1
	People working in public involvement in research	1
Stakeholders	Religious and community leaders	1
	Parents	2
	Teachers	3
	Other stakeholders	6
Young people	Adolescents	11
	Peer educators	1

**Table 3:** Terminologies used to refer to adolescent involvement in the included guidelines.

Terminologies	*n*
Participation	20
Involve	19
Engage	18
Partnership	6
Participatory action research	6
Youth‐led	4
Co‐research	4
Peer education	4
Public patient involvement	3
Peer‐based programmes	3
Co‐production	3
Approach not reported	3
Participatory evaluation	3
Inclusion	2
Participatory	2
Youth Advisory Board	2
Working with youth	1
Youth Advisory Councils	1
Sponsorship	1
Consultation	1
Child Centred Community Development	1
Youth centred	1
Youth mainstreaming	1

### 3.3 Content of the guidelines

In total, the 88 guidelines covered 45 topics pertaining to adolescent involvement in health research (Table 4). These topics were discerned through a careful analysis of the data extracted from the included guidelines. The extracted data was coded, and relevant codes were merged under broader themes around key aspects of adolescent involvement. This process resulted in 45 themes or topics on different aspects of adolescent involvement in health research. However, none of the included guidelines included all 45 topics. The most comprehensive set of guidelines covered 20 out of the total 45 topics.^40^, ^41^, ^65^ This indicates that even the most detailed guidelines fell short of encompassing more than half of the important areas regarding adolescent involvement in research (Supplementary Table 2). On average, each set of guidelines included 6.41 topics. The most frequently included topics across guidelines included the type of adolescent involvement approach (*n* = 55), identifying the target population and their recruitment (*n* = 37), and involving adolescents at different stages of the project (*n* = 32).

**Table 4:** Number of guidelines covering specific topics relevant to adolescent involvement in health research.

Topics covered in guidelines	Moderate to extensive details provided	Very limited information included	Total number of guidelines covering this topic
Defining the approach used	36	19	55
Identifying target population and recruitment	23	14	37
Adolescent involvement at different stages of the research project	24	8	32
Preparation and planning for involving adolescents in health research	20	12	32
Model used	14	12	26
Training	11	15	26
Monitoring and Evaluation	18	7	25
Meaningful involvement of adolescents	9	14	23
Ethics, safety, and protection	11	11	22
Principles of ethical involvement	6	16	22
Barriers	19	2	21
Reimbursement	12	8	20
Characteristics and roles of adults in adolescent involvement	10	8	18
Training the assessment team	12	5	17
Level of adolescent involvement	12	5	17
Informed consent	11	6	17
Conducting meetings and workshops with adolescents	11	6	17
Inclusiveness and accessibility	8	7	15
Creating an enabling environment	7	8	15
Engaging other stakeholders	4	10	14
Flexibility	3	11	14
Methods to engage adolescents	4	7	11
Power dynamics	2	9	11
Peer education—training of trainers	5	5	10
Communication between meetings or activities	3	5	8
Training adolescents to be researchers	4	0	4
Overall things adolescents should know about when participating in health research projects	4	0	4
Making research enjoyable	2	2	4
Adolescents' exit from programmes	0	4	4
Embedding participation within an organisation	2	1	3
Overall training on monitoring and evaluation	2	0	2
Activities to use in workshops	2	0	2
What are the gaps or future directions in adolescent engagement work?	2	0	2
Tools to use in adolescent involvement	2	0	2
Data protection	1	0	1
Participatory assessment	1	0	1
Involving adolescent to conduct events	1	0	1
Ensuring everyone's input	1	0	1
Reporting adolescent engagement	1	0	1
Getting started with an adolescent participation strategy in an organisation	1	0	1
Health‐area specific suggestions	1	0	1
Child‐led data collection	1	0	1
Involving vulnerable adolescents	1	0	1
Exiting the field	0	1	1
Lessons learned and general recommendations for adolescent involvement	0	1	1

### 3.4 Context of the guidelines

The countries in which the guidelines were developed were determined based on the regions where stakeholders were involved and the locations of the organisations leading the guideline development.

#### 3.4.1 Stakeholder involvement

We extracted data on the countries in which stakeholders—including adolescents, professionals, and other researchers with expertise in adolescent involvement—participated in the guideline development process. Only 33 guidelines reported the countries where stakeholders were engaged. Of these, 15% (*n* = 13) involved stakeholders from high‐income countries, 9% (*n* = 8) from low‐ and middle‐income countries, 10% (*n* = 9) from across all three country types, that is, high‐, and low‐ and middle‐income countries, and 3% (*n* = 3) only reported the overall regions where stakeholders were involved.

#### 3.4.2 Organisations involved in the development of guidelines

To ascertain the countries where the guidelines were developed, the locations of the involved organisations were also considered. In the case of international organisations, the location of the country office participating in the guideline development was used to determine the country. In instances where authors were affiliated with different universities or organisations in different countries, all relevant countries were included as locations where the guidelines were developed. Based on this, 72% of guidelines (*n* = 63) were developed by organisations in high‐income countries, 3% (*n* = 3) by organisations in low‐ and middle‐income countries, and 16% (*n* = 14) by organisations across all three country types, that is, high‐, and low‐ and middle‐income countries. The organisations involved in the development of guidelines were not reported for 5% (*n* = 4) of the guidelines, while 5% of guidelines (*n* = 4) reported only the region of the organisations instead of the specific countries.

Most guidelines were authored by researchers from universities (*n* = 24), Save the Children (*n* = 10), International Planned Parenthood Federation (*n* = 5), and UNICEF (*n* = 4). The highest number of organisations contributing to a single set of guidelines was seven.^66^ Out of the included guidelines, 22 provided descriptions of the specific contexts in which they are applicable. These contexts varied and included humanitarian settings (*n* = 2), organisational settings (*n* = 2), schools (*n* = 2), community settings (*n* = 5), the health sector (*n* = 2), virtual research (*n* = 3), and research in all contexts (*n* = 6).

### 3.5 Quality of guidelines

The quality of guidelines varied significantly across the six AGREE‐II domains (Table 5, Supporting Information S1: Table 3). Most guidelines performed well in Domain 1 (scope and purpose), with 44% achieving scaled domain scores above 50%. This suggests guidelines provided clear objectives and clearly defined health areas and target populations. However, in Domain 2 (stakeholder involvement), 68% of the guidelines scored less than 50% on the scaled domain score due to insufficient engagement with relevant stakeholders—including adolescents—and/or because they provided unclear descriptions of target users. Domain 3 (rigour of development) consistently received low scores, with 99% of the guidelines having scaled domain scores of less than 50%. This indicates a lack of systematic methods in guideline development. For Domain 4 (clarity of presentation), 42% of the guidelines achieved scaled domain scores above 50%. Recommendations were generally presented clearly, and key points were easily identifiable in most guidelines. Guidelines performed poorly on Domain 5 (applicability of the recommendations), with 93% scoring less than 50% on the scaled domain score. This was attributed to inadequate consideration of resources, barriers, and monitoring criteria, suggesting a lack of practical guidance for implementation. Lastly, guidelines performed particularly poorly on Domain 6 (editorial independence), with 98% scoring less than 50% on the scaled domain score. This highlights a lack of clarity concerning conflicts of interest and the involvement of funding bodies in guideline development.

**Table 5:** Quality assessment of included guidelines.

Domains	Items	Strongly disagree	Somewhat agree	Strongly agree
Domain 1. Scope and purpose	1. The overall objective(s) of the guideline is (are) specifically described.	10 (11.4%)	9 (10.2%)	69 (78.4%)
	2. The health question(s) covered by the guideline is (are) specifically described.	33 (37.5%)	11 (12.5%)	44 (50.0%)
	3. The population (patients, public, etc.) to whom the guideline is meant to apply is specifically described.	52 (59.1%)	7 (8.0%)	29 (33.0%)
Domain 2. Stakeholder involvement	4. The guideline development group includes individuals from all relevant professional groups.	50 (56.8%)	32 (36.4%)	6 (6.8%)
	5. The views and preferences of the target population (patients, public, etc.) have been sought.	49 (55.7%)	11 (12.5%)	28 (31.8%)
	6. The target users of the guideline are clearly defined.	48 (54.5%)	9 (10.2%)	31 (35.2%)
Domain 3. Rigour of development	7. Systematic methods were used to search for evidence.	71 (80.7%)	12 (13.6%)	5 (5.7%)
	8. The criteria for selecting the evidence are clearly described.	84 (95.5%)	3 (3.4%)	1 (1.1%)
	9. The strengths and limitations of the body of evidence are clearly described.	87 (98.9%)	1 (1.1%)	0 (0.0%)
	10. The methods for formulating the recommendations are clearly described.	32 (36.4%)	36 (40.9%)	20 (22.7%)
	11. The health benefits, side effects, and risks have been considered in formulating the recommendations.	69 (78.4%)	15 (17.0%)	0 (0.0%)
	12. There is an explicit link between the recommendations and the supporting evidence.	88 (100.0%)	0 (0.0%)	0 (0.0%)
	13. The guideline has been externally reviewed by experts before its publication.	61 (69.3%)	8 (9.1%)	19 (21.6%)
	14. A procedure for updating the guideline is provided.	82 (93.2%)	3 (3.4%)	3 (3.4%)
Domain 4. Clarity of presentation	15. The recommendations are specific and unambiguous.	10 (11.4%)	46 (52.3%)	32 (36.4%)
	16. Key recommendations are easily identifiable.	15 (17.0%)	43 (48.9%)	30 (34.1%)
Domain 5. Applicability	17. The guideline describes facilitators and barriers to its application.	68 (77.3%)	15 (17.0%)	5 (5.7%)
	18. The guideline provides advice and/or tools on how the recommendations can be put into practice.	25 (28.4%)	36 (40.9%)	27 (30.7%)
	19. The potential resource implications of applying the recommendations have been considered.	72 (81.8%)	15 (17.0%)	1 (1.1%)
	20. The guideline presents monitoring and/or auditing criteria.	74 (84.1%)	12 (13.6%)	2 (2.3%)
Domain 6. Editorial independence	21. The views of the funding body have not influenced the content of the guideline.	81 (92.0%)	1 (1.1%)	6 (6.8%)
	22. Competing interests of guideline development group members have been recorded and addressed.	83 (94.3%)	0 (0.0%)	5 (5.7%)

## 4 DISCUSSION

This review provides the most comprehensive list of guidance on adolescent involvement in health research, identifies limitations of the available guidelines, and suggests directions for future research to develop more comprehensive, and rigorous guidelines by addressing these limitations. We found that the currently available guidelines on adolescent involvement are limited in scope and lack comprehensive coverage of recommendations for adolescent involvement in health research. Many were developed for specific contexts and sectors, which limits their applicability. Furthermore, a significant proportion of these guidelines are of low quality, which raises concerns about their effectiveness in guiding meaningful and impactful adolescent involvement in health research.

### 4.1 Scope of the guidelines

While many guidelines were centred around the overall involvement and participation of adolescents, no single guideline comprehensively covered all the different types of adolescent involvement methods or provided guidance on when to use each approach. This is an important limitation because previous research has highlighted the strong need for guidance on how to choose the appropriate approach or model of adolescent involvement from the available approaches and methods.^6^, ^67^, ^68^ Guidelines developed in future should incorporate guidance on different adolescent involvement approaches to help researchers select the appropriate methodology for their project.

### 4.2 Content of the guidelines

The highest number of topics covered in a single set of guidelines was 20 out of a total of 45 topics covered across all included guidelines. This indicates that none of the guidelines were comprehensive in terms of the content covered on adolescent involvement in health research. While all 45 topics related to adolescent involvement covered across the included guidelines may not be essential for inclusion in future guidelines, there are several key areas that should be prioritised. These key areas are recruitment, retention, preparation and planning for adolescent involvement, and training of both researchers and adolescents. These topics should be considered essential components of all guidelines on adolescent involvement. The current limitations in coverage could potentially lead to oversights and gaps in understanding, thereby hindering meaningful involvement of adolescents in health research. These findings align with Wilson et al.'s review,^6^ and emphasise the need for a more comprehensive set of guidelines that cover all key aspects of adolescent involvement. Furthermore, most of the included topics were not covered in detail and were discussed as generic principles. In other words, most guidelines provided a brief overview of what should be done, rather than delving into the specifics of how it should be done. This is problematic because researchers often struggle with applying generic guidance on involvement^68^ and implementing the overall concepts of adolescent involvement in practice.^68^, ^69^, ^70^ To address this, future guidelines need to provide strategies or guidance for how to operationalise generic principles of adolescent involvement for different target populations and contexts.

### 4.3 Context of the guidelines

Most of the included guidelines originated from four high‐income countries, which is consistent with the predominant focus of adolescent involvement in health research within these countries.^6^, ^8^ Despite approximately 90% of adolescents residing in low‐ and middle‐income countries,^1^ only 19% of the guidelines were developed in consultation with stakeholders or organisations from these countries. This discrepancy poses a significant limitation in the broad applicability of these guidelines, emphasising the urgent need for the development of more resources that are applicable in low‐ and middle‐income countries.

Among the guidelines providing information on specific applicable contexts, only six were reported to be universally applicable. This suggests that most guidelines are tailored for specific circumstances, limiting their universal relevance. It is widely acknowledged that frameworks and guidelines developed for specific contexts are often challenging to transfer to other settings, as different settings pose unique challenges.^71^

### 4.4 Quality of the guidelines

The quality was compromised by a lack of rigorous development methods and low applicability of recommendations in real‐world settings. In terms of development methods used, guidelines generally fell into two categories: those based on evidence from the literature and those grounded in practical experience, such as insights from professionals, adolescents, and other stakeholders. Concerningly, only 20 guidelines were based on some form of literature review, contrary to recommended guideline development practices.^72^ This raises questions about the robustness of evidence supporting most guidelines. In terms of practice‐based evidence, only 28 guidelines were grounded in previous research projects or guidelines, less than half of the guidelines involved stakeholders other than adolescents, and only 58% involved adolescents. Although the level of adolescent involvement can vary based on the context and logistical restrictions, it is crucial for guideline developers to at least consult adolescents in the guideline development process by seeking and incorporating their perspectives and feedback. Without this, the development of the guidelines for adolescent involvement will be happening in a way which runs counter to the goals of those guidelines themselves. The absence of diverse perspectives in the guideline development process may compromise the applicability and relevance of the guidelines. To enhance the robustness of future guidelines, it is imperative to prioritise inclusive stakeholder involvement throughout the development process. Adolescents were often engaged at the lower rungs of the involvement ladder, limiting their role to providing feedback and consultations at the later stages of the guideline development process. This indicates that adolescents had limited involvement and input in setting the agenda and shaping the content of most available guidelines, which goes against the core principles of public and patient involvement of adolescents.

Ninety‐three percent of the guidelines received low scores for their applicability in the real‐world settings as they provided limited information on implementation‐related factors such as resources needed to implement the recommendations in practice and the barriers and facilitators to different recommendations. This finding is consistent with existing literature on adolescent involvement, which notes that the currently available guidelines on adolescent involvement often offer principles rather than actionable and operationalised instructions on how to implement these principles.^19^ Given that a major contributing factor to the limited involvement of adolescents in health research is the lack of awareness among researchers on how to involve adolescents and address associated barriers,^4^, ^6^, ^18^ it is crucial for guidelines to prioritise applicability and provide clear operationalised recommendations.

### 4.5 Comparison with other studies

To the best of our knowledge, this is the first review of the currently available guidelines on adolescent involvement in health research. In the domain of overall public and patient involvement, a few studies have consolidated current guidance with a special focus on models and frameworks. For instance, Greenhalgh et al.^71^ provided a taxonomy of existing frameworks of public and patient involvement based on their focus and objectives. Harrison et al.^73^ synthesised available frameworks and guidelines to summarise principles, best practices, and stages of research where the public should be involved. Jull et al.^74^ and Chudyk et al.^75^ provided syntheses of the core concepts and underlying elements presented in frameworks of public and patient involvement. However, none of these reviews evaluated the scope and quality of the included frameworks and guidance. The syntheses of frameworks and guidelines on overall public and patient involvement concluded that a one‐size‐fits‐all framework would not be appropriate, and users should have a toolbox of evidence‐based resources that they can choose from based on their resources, project, and team structure, as indicated by Greenhalgh et al.^71^ and Chudyk et al.^75^ Our review builds on this recommendation and provides a comprehensive list and evaluation of available guidelines. This review furthers the conversation on what topics should be considered ‘essential’ as part of any guideline on adolescent involvement in research.

### 4.6 Future directions for research

Several common elements found in the included guidelines serve as starting points for developing a baseline set of quality requirements, particularly related to development methodology and content. Regarding development methodology, future guidelines should be grounded in rigorously conducted literature reviews, draw upon existing resources, and involve a diverse range of stakeholders, with a particular emphasis on engaging adolescents in the guideline development process. The developed guidelines should undergo piloting and review by independent experts, particularly those with expertise in adolescent involvement in research.

Regarding content, the guidelines should cover some key areas of adolescent involvement outlined below. These include defining various approaches to adolescent involvement, identifying target populations and recruitment strategies, detailing methods for involving adolescents at different stages of the research project, outlining preparation and planning for involving adolescents in health research, training adolescents and researchers, ethical involvement of adolescents and ensuring their safety, best practices of adolescent involvement, engaging other stakeholders, and different methods for engaging adolescents in research.

Overall, there is a need for a comprehensive set of guidelines that encompass all key topics of adolescent involvement while being flexible enough to allow adaptation of these guidelines for different contexts, users, and areas of health research. While a rigid one‐size‐fits‐all approach to guidelines may not be suitable for all stakeholders and contexts, there is value in establishing comprehensive guidance that can be adapted as needed.

## 5 CONCLUSION

This review presents a comprehensive synthesis and evaluation of available guidance on involving adolescents in health research. To improve the meaningful engagement of adolescents in health research, it is imperative to address the limitations in current guidance. There is a clear demand for more evidence‐based and comprehensive guidelines that are adaptable for different contexts and users. The development of future guidelines should prioritise collaborative efforts between researchers and stakeholders, ensuring an inclusive approach towards adolescent involvement in health research.

## AUTHOR CONTRIBUTIONS

**Azza Warraitch**: Conceptualisation; investigation; funding acquisition; writing—original draft; methodology; validation; writing—review and editing; formal analysis; software; data curation. **Ciara Wacker**: Methodology; writing—review and editing; data curation; investigation. **Delali Bruce**: Investigation; writing—review and editing; methodology; data curation. **Ashling Bourke**: Investigation; methodology; writing ‐ review and editing. **Kristin Hadfield**: Conceptualisation; investigation; funding acquisition; writing—review and editing; methodology; project administration; supervision.

## CONFLICT OF INTEREST STATEMENT

The authors declare no conflict of interest.

## Supporting information

Supplementary information.

## Data Availability

The data that support the findings of this study are openly available in Open Science Framework at https://osf.io/au6dh/?view_only=621932b8ee274fa5b85876b92cf62e7a, reference number doi:10.17605/OSF.IO/AU6DH.
