# Supplementary material for: A rapid review of guidelines on the involvement of adolescents in health research
Source: Health Expect. 2024 Jun 10;27(3):e14058. doi: 10.1111/hex.14058 (PMC11163265; doi:10.1111/hex.14058)
Supplement: Supplementary file 1 — Supplementary information. [file HEX-27-e14058-s001.docx]

**Supplementary Figure 1**

*Year of Publication of the Included Guidelines*

**
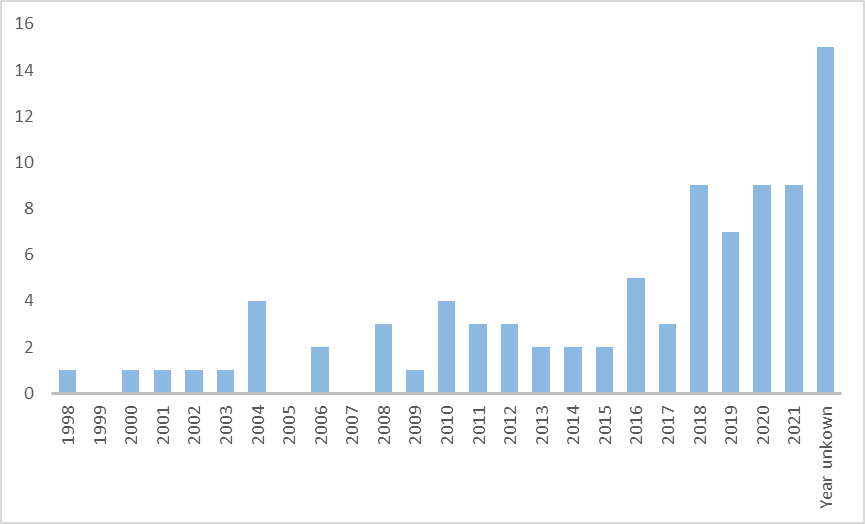
**

**Supplementary Table 1**

*Search strategy for CINAHL*

| **#** | **Query** | **Limiters/Expanders** | **Last Run Via** | **Results** |
| --- | --- | --- | --- | --- |
| S12 | S1 AND S5 AND S10 | Expanders - Apply equivalent subjects Narrow by Language: - english Search modes - Boolean/Phrase | Interface - EBSCOhost Research Databases Search Screen - Advanced Search Database - CINAHL Complete | 649 |
| S11 | S1 AND S5 AND S10 | Expanders - Apply equivalent subjects Search modes - Boolean/Phrase | Interface - EBSCOhost Research Databases Search Screen - Advanced Search Database - CINAHL Complete | 657 |
| S10 | S6 OR S7 OR S8 OR S9 | Expanders - Apply equivalent subjects Search modes - Boolean/Phrase | Interface - EBSCOhost Research Databases Search Screen - Advanced Search Database - CINAHL Complete | 33,218 |
| S9 | (MM "Consumer Participation") | Expanders - Apply equivalent subjects Search modes - Boolean/Phrase | Interface - EBSCOhost Research Databases Search Screen - Advanced Search Database - CINAHL Complete | 12,825 |
| S8 | (MM "Stakeholder Participation") | Expanders - Apply equivalent subjects Search modes - Boolean/Phrase | Interface - EBSCOhost Research Databases Search Screen - Advanced Search Database - CINAHL Complete | 461 |
| S7 | (MM "Action Research") | Expanders - Apply equivalent subjects Search modes - Boolean/Phrase | Interface - EBSCOhost Research Databases Search Screen - Advanced Search Database - CINAHL Complete | 840 |
| S6 | TI ( advisory* OR "youth engagement" OR "patient and public involvement" OR "public and patient involvement" OR "adolescent engagement" OR "participatory*" OR "Human centered design" OR "Human centred design" OR "User centered design" OR "User centred design" ) OR AB ( advisory* OR "youth engagement" OR "patient and public involvement" OR "public and patient involvement" OR "adolescent engagement" OR "participatory*" OR "Human centered design" OR "Human centred design" OR "User centered design" OR "User centred design" ) | Expanders - Apply equivalent subjects Search modes - Boolean/Phrase | Interface - EBSCOhost Research Databases Search Screen - Advanced Search Database - CINAHL Complete | 20,648 |
| S5 | S2 OR S3 OR S4 | Expanders - Apply equivalent subjects Search modes - Boolean/Phrase | Interface - EBSCOhost Research Databases Search Screen - Advanced Search Database - CINAHL Complete | 229,928 |
| S4 | (MM "Adolescence") | Expanders - Apply equivalent subjects Search modes - Boolean/Phrase | Interface - EBSCOhost Research Databases Search Screen - Advanced Search Database - CINAHL Complete | 1,586 |
| S3 | (MM "Young Adult") | Expanders - Apply equivalent subjects Search modes - Boolean/Phrase | Interface - EBSCOhost Research Databases Search Screen - Advanced Search Database - CINAHL Complete | 367 |
| S2 | TI ( youth OR adolescen* OR "young people" OR "Young person*" OR "Young adult*" ) OR AB ( youth OR adolescen* OR "young people" OR "Young person*" OR "Young adult*" ) | Expanders - Apply equivalent subjects Search modes - Boolean/Phrase | Interface - EBSCOhost Research Databases Search Screen - Advanced Search Database - CINAHL Complete | 229,102 |
| S1 | TI ( Guid* OR recommendation* OR framework OR toolkit ) OR AB ( Guid* OR recommendation* OR framework OR toolkit ) | Expanders - Apply equivalent subjects Search modes - Boolean/Phrase | Interface - EBSCOhost Research Databases Search Screen - Advanced Search Database - CINAHL Complete | 523,266 |

**Supplementary Table 2**

*List of Guidelines Included in the Rapid Review and Number of Relevant Youth Involvement Topics Included in All Guidelines.*

| **IDs of Guidelines** | **Link** |  | **Name of guidelines** | **Moderate to extensive details provided** | **Very limited information included** | **Total numb-er of topics covered in this guideline** |
| --- | --- | --- | --- | --- | --- | --- |
| 1 | <https://cdn.iawg.rygn.io/documents/ASRH%20Toolkit%202020%20Edition/English/ASRH%20Toolkit/Ch3%20uploads/IAWG-Toolkit_Chapter-3.pdf?mtime=20210719202756&focal=none> |  | Chapter 3: meaningful participation | 7 | 8 | 15 |
| 2 | <https://www.ippf.org/sites/default/files/inspire_explore.pdf> |  | Explore: ideas for youth involvement in research | 8 | 3 | 11 |
| 3 | <https://rutgers.international/resources/explore-toolkit-instructions/> |  | Toolkit for involving young people as researchers in sexual and reproductive health programmes | 10 | 4 | 14 |
| 4 | <https://ypeerap.org/wp-content/uploads/2020/03/peer_evidence-Based-_guidelines1-1.pdf> |  | Evidence-based guidelines for youth peer education | 10 | 4 | 14 |
| 5 | <https://www.cacap-acpea.org/wp-content/uploads/Recommendations-for-youth-engagement-in-Canadian-mental.pdf> |  | Recommendations for youth engagement in Canadian mental health research in the context of COVID-19 | 0 | 1 | 1 |
| 6 | <https://www.invo.org.uk/wp-content/uploads/2012/01/InvolvingYoungPeople2004.pdf> |  | A guide to actively involving young people in research | 15 | 5 | 20 |
| 7 | <https://www.invo.org.uk/wp-content/uploads/2016/05/CYP-reward-and-recognition-Final-April2016.pdf> |  | Reward and recognition for children and young people involved in research – things to consider | 1 | 1 | 2 |
| 8 | <https://mcpin.org/wp-content/uploads/2021/12/Involving-young-people-in-research-work-guide.pdf> |  | 10 things everyone needs to know about running a meeting with young people | 9 | 0 | 9 |
| 9 | <https://www.wvi.org/sites/default/files/WV_Guidelines_on_Child_Participation.pdf> |  | World vision’s guidelines for child participation | 5 | 3 | 8 |
| 10 | [file:///C:/Users/awarr/Downloads/aye_mena-toolkit%20(2).pdf](file:///C:\Users\awarr\AppData\Roaming\Users\awarr\Downloads\aye_mena-toolkit%20(2).pdf) |  | Toolkit for adolescent and youth engagement | 8 | 6 | 14 |
| 11 | <https://rutgers.international/resources/explore-training-manual/> |  | Explore: training manual for monitoring & evaluation and research for young people | 3 | 0 | 3 |
| 12 | <https://generationr.org.uk/wp-content/uploads/2018/06/Health-Research-Authority-Staff-Day26April18.pdf> |  | Creating a space for young people’s involvement in health research | 2 | 1 | 3 |
| 13 | <https://dera.ioe.ac.uk/17522/1/Handbook%20-%20Building%20a%20Culture%20of%20Participation.pdf> |  | Building a culture of participation | 1 | 1 | 2 |
| 14 | <https://www.invo.org.uk/wp-content/uploads/2016/01/involvingcyp-top-tips-January2016.pdf> |  | Involving children and young people in research: top tips for researchers | 3 | 6 | 9 |
| 15 | <https://www.ncb.org.uk/sites/default/files/uploads/files/PEAR%2520guidelines.pdf> |  | Young people in research: how to involve us guidance for researchers from the pear young people’s public health group | 3 | 11 | 14 |
| 16 | <https://www.rcpch.ac.uk/sites/default/files/generated-pdf/document/How-to-write-a-children-and-young-people%2527s-engagement-plan---RCPCH-%2526Us.pdf> |  | How to write a children and young people's engagement plan - RCPCH &us | 2 | 0 | 2 |
| 17 | <https://www.lshtm.ac.uk/sites/default/files/2019-06/Consulting-Children-Young-People-Disabilities.pdf> |  | Guidelines for consulting with children & young people with disabilities | 1 | 3 | 4 |
| 18 | <https://www.orygen.org.au/About/Youth-Engagement/Resources/YouthPartnershipToolkit.aspx> |  | How to partner with young people | 1 | 1 | 2 |
| 19 | <https://www.orygen.org.au/About/Youth-Engagement/Resources/youth-peer-work-toolkit.aspx> |  | What is peer work? | 0 | 1 | 1 |
| 20 | <https://orygen.org.au/Training/Resources/Depression/Toolkits/Including-student-voices-in-school-based-mental-he/orygen-schools_Toolkit_Student-voice-pdf.aspx?ext=.> |  | Including student voice in school-based mental health programs | 1 | 1 | 2 |
| 21 | https://pdf.usaid.gov/pdf_docs/PNACK128.pdf |  | Involving youth in reproductive health projects 1998 | 6 | 8 | 14 |
| 22 | https://foundrybc.ca/wp-content/uploads/2020/06/E.12-INNOVATE-Research-Youth-Engagement-Guidebook.pdf |  | Innovate research youth engagement guidebook for researchers | 12 | 8 | 20 |
| 23 | https://ypeerap.org/kiribati/wp-content/uploads/2020/05/Y-PEER-TOT-Manual-Orginal-8-4.pdf |  | Youth peer education toolkit, training of trainers’ manual | 3 | 0 | 3 |
| 24 | https://www.youthpower.org/sites/default/files/YouthPower/resources/Brief_4_FINAL_edited_2-17%20pdf.pdf |  | Six tips for increasing meaningful youth engagement in programs | 3 | 1 | 4 |
| 25 | https://www.ippf.org/sites/default/files/2018-08/Young-people-at-the-centre-May17.pdf |  | Vision and plan for placing young people at the heart of implementing IPPF’s strategic framework | 1 | 0 | 1 |
| 26 | https://resourcecentre.savethechildren.net/pdf/child_friendly_par_toolkit_small.pdf/ |  | Child- and youth-friendly participatory action research toolkit | 1 | 0 | 1 |
| 27 | https://www.youngminds.org.uk/media/nf5h02mx/ym-amplified-digital-participation-toolkit.pdf |  | Digital engagement in participation | 2 | 0 | 2 |
| 28 | https://resourcecentre.savethechildren.net/pdf/7191.pdf/ |  | Guide on participatory monitoring and evaluation methodologies for working with children and youth – SOS children’s villages | 12 | 7 | 19 |
| 29 | https://resourcecentre.savethechildren.net/pdf/5750.pdf/ |  | The European union and child participation | 0 | 7 | 7 |
| 30 | <https://resourcecentre.savethechildren.net/pdf/toolbox_of_participative_tools.pdf/> |  | Toolbox of participative tools: adapted from the UNICEF workshop on participatory approaches for working with children and young people in programme planning, implementation and studies and evaluation | 4 | 0 | 4 |
| 31 | <https://info.lse.ac.uk/staff/divisions/research-and-innovation/research/Assets/Documents/PDF/NCB-guidelinesCYP-2011.pdf> |  | Guidelines for research with children and young people | 10 | 3 | 13 |
| 32 | https://www.unodc.org/res/prevention/youth-initiative/resources-new_html/Handbook_on_Youth_Participation.pdf |  | Youth participation in drug prevention work | 6 | 7 | 13 |
| 33 | https://www.unodc.org/pdf/youthnet/equal_partners_guide.pdf |  | Equal partners organizing for youth-by-youth events | 1 | 1 | 2 |
| 34 | https://www.youngminds.org.uk/media/a5idx2ji/what-is-participation.pdf |  | What is participation in children and young people's mental health? | 2 | 5 | 7 |
| 35 | https://tciurbanhealth.org/courses/philippines-toolkit-youth-engagement/lessons/meaningful-youth-engagement/ |  | Philippines toolkit: youth engagement | 4 | 3 | 7 |
| 36 | file:///C:/Users/awarr/Downloads/NigerianGuideline.pdf |  | Guidelines for young persons’ participation in research and access to sexual and reproductive health services in Nigeria | 1 | 8 | 9 |
| 37 | https://archives.studentscommission.ca/sandbox-roundtable/offline/download.pdf |  | Promising practices from the sandbox project's young Canadians roundtable on health | 0 | 1 | 1 |
| 38 | https://tciurbanhealth.org/courses/east-africa-advocacy/lessons/youth-engagement/ |  | East Africa toolkit: ASYRH advocacy | 0 | 6 | 6 |
| 39 | https://irp-cdn.multiscreensite.com/29f8e96e/files/uploaded/Ethics%20in%20YP%20participation%20and%20research%20CommonRoom.pdf |  | How to manage research and participation projects: the ethics according to young people | 1 | 7 | 8 |
| 40 | http://mypeer.org.au/planning/what-are-peer-based-programs/how-do-peer-based-programs-work-2/ |  | [My-peer toolkit](http://mypeer.org.au/planning/what-are-peer-based-programs/how-do-peer-based-programs-work-2/) | 3 | 0 | 3 |
| 41 | https://resourcecentre.savethechildren.net/pdf/par_lessons_learned_report_how_to_guide.pdf/ |  | Youth voices participatory action research with adolescents affected by the Syria crisis in Egypt and Lebanon lessons learned report and ‘how to’ guide | 12 | 3 | 15 |
| 42 | https://eppi.ioe.ac.uk/cms/Default.aspx?tabid=3681&articleType=ArticleView&articleId=175 |  | [Involving young people with lived experience of adverse childhood experience (aces) in a systematic review](https://eppi.ioe.ac.uk/cms/Default.aspx?tabid=3681&articleType=ArticleView&articleId=175) | 1 | 1 | 2 |
| 43 | https://www.who.int/publications/i/item/9789240011717 |  | Youth-centred digital health interventions: a framework for planning, developing and implementing solutions with and for young people | 4 | 10 | 14 |
| 44 | https://3532bf5a-d879-4481-8c8f-127da8c44deb.usrfiles.com/ugd/3532bf_d03284e2369945c09608b9736c381d1e.pdf |  | Involving young Londoners: a toolkit for peer research | 11 | 1 | 12 |
| 45 | https://generationr.org.uk/virtualcopro/ |  | Exploring co-production in a virtual world – what we’re learning | 1 | 0 | 1 |
| 46 | https://www.learningforinvolvement.org.uk/wp-content/uploads/2021/04/NIHR-Involving-children-and-young-people-as-advisors-in-research-April-2021.pdf |  | Involving children and young people as advisors in research top tips and essential key issues for researchers | 10 | 10 | 20 |
| 47 | https://participationpool.eu/wp-content/uploads/2020/07/Youth-as-Researchers-Training-Manual.pdf |  | Youth as researchers training manual | 1 | 0 | 1 |
| 48 | https://esaro.unfpa.org/sites/default/files/pub-pdf/GUIDANCE%20ON%20ENHANCING%20YOUTH%20PARTICIPATION%20IN%20ESA.pdf |  | Guidance on enhancing youth participation in ESA | 5 | 2 | 7 |
| 49 | https://www.advocatesforyouth.org/resources/fact-sheets/building-effective-youth-adult-partnerships/?option=com_content&task=view&id=673&Itemid=336 |  | Building effective youth-adult partnerships | 2 | 1 | 3 |
| 50 | https://eypagnet.eu/wp-content/uploads/2020/11/the_evaluators_cookbook_participatory_evaluation_exercises_for_young_people.pdf |  | The evaluator’s cookbook participatory evaluation exercises a resource for work with children and young people | 1 | 0 | 1 |
| 51 | https://www.participatorymethods.org/sites/participatorymethods.org/files/participatory%20evaluation%20with%20young%20people_Checkoway.pdf |  | Participatory evaluation with young people | 2 | 1 | 3 |
| 52 | file:///C:/Users/awarr/Downloads/Youth%20Changing%20the%20World%20Toolkit.pdf |  | Youth changing the world | 2 | 0 | 2 |
| 53 | https://www.icanresearch.org/ican-curriculum |  | ICAN curriculum | 2 | 0 | 2 |
| 54 | https://drive.google.com/file/d/1aKZ69G64nYqeE-kd_tE0I9yY7v8E_zO8/view |  | What is co-research? | 7 | 1 | 8 |
| 55 | https://eypagnet.eu/toolkit/ |  | Generation r alliance/eYPAGNET toolkit | 8 | 5 | 13 |
| 56 | https://opa.hhs.gov/sites/default/files/2020-10/OPA_Youth_Toolkit_Final_508.pdf |  | Youth listening session toolkit | 1 | 0 | 1 |
| 57 | https://www.sciencedirect.com/science/article/pii/S0190740918304614 |  | Re-conceptualising youth participation: a framework to inform action | 3 | 1 | 4 |
| 58 | https://adc.bmj.com/content/archdischild/105/9/875.full.pdf?casa_token=jDcWnDu_VdYAAAAA:cJsHeE9wXy-tggk9KrAuF1pAyKmiGbJDY5r17HZ_NlI4astUfDrqXGfAl5j7UZUoAlVXo92aHg |  | Guidance on development and operation of young persons’ advisory groups | 2 | 0 | 2 |
| 59 | https://onlinelibrary.wiley.com/doi/pdf/10.1111/j.1099-0860.2006.00062.x?casa_token=MNae-6OMESgAAAAA:tmMYa4o96o4DSDQs0dyYsL6Hk_Aedn26jTysS3oJE4m1Mn3StdNKpUSPvHv9s3LONa2X9yW-YBSWNkk |  | Reflections on practical approaches to involving children and young people in the data analysis process | 1 | 0 | 1 |
| 60 | https://onlinelibrary.wiley.com/doi/full/10.1111/hex.12795 |  | Engaging youth in research planning, design and execution: practical recommendations for researchers | 4 | 7 | 11 |
| 61 | https://academic.oup.com/intqhc/article/31/2/147/4999232?login=true |  | Involving young people in health promotion, research and policymaking: practical recommendations | 4 | 11 | 15 |
| 62 | https://www.tandfonline.com/doi/full/10.1080/01944363.2019.1616319 |  | Including youth in the ladder of citizen participation | 1 | 0 | 1 |
| 63 | http://www.reservoircg.org/uploads/2/9/1/1/2911062/par_and_tay.pdf |  | Participatory action research and young adults with psychiatric disabilities | 2 | 0 | 2 |
| 64 | https://ep.bmj.com/content/97/2/55.long |  | ‘Nothing about us without us’: considerations for research involving young people | 2 | 6 | 8 |
| 65 | https://files.eric.ed.gov/fulltext/EJ1095954.pdf |  | Measuring and understanding authentic youth engagement the youth-adult partnership rubric | 2 | 0 | 2 |
| 66 | https://onlinelibrary.wiley.com/doi/full/10.1007/s10464-010-9330-0 |  | A typology of youth participation and empowerment for child and adolescent health promotion | 1 | 0 | 1 |
| 67 | https://www.jstor.org/stable/pdf/26734126.pdf?refreqid=excelsior%3A8fe992d4d9746129c726b772974c712a&ab_segments=&origin=&initiator=&acceptTC=1 |  | Teens as advocates for substance use prevention: strategies for implementation | 2 | 6 | 8 |
| 68 | https://onlinelibrary.wiley.com/doi/pdf/10.1002/yd.312 |  | From voice to agency: guiding principles for participatory action research with youth | 1 | 0 | 1 |
| 69 | https://www.ncbi.nlm.nih.gov/pmc/articles/PMC6075896/ |  | Nothing about us without rights—meaningful engagement of children and youth: from research prioritization to clinical trials, implementation science, and policy | 1 | 7 | 8 |
| 70 | https://ep.bmj.com/content/104/4/195 |  | Ethics and patient and public involvement with children and young people | 2 | 11 | 13 |
| 71 | https://www.tandfonline.com/doi/full/10.1080/0145935X.2014.962132 |  | Youth-guided youth engagement: participatory action research (par) with high-risk, marginalized youth | 1 | 0 | 1 |
| 72 | https://www.fhi360.org/sites/default/files/media/documents/Engaging%20Communities%20in%20Youth%20Reproductive%20Health.pdf |  | Engaging communities in youth reproductive health and HIV projects a guide to participatory assessments | 4 | 4 | 8 |
| 73 | https://resourcecentre.savethechildren.net/pdf/5901.pdf/ |  | Child led data collection: a guide for young people to learn how to do research and create positive change | 2 | 0 | 2 |
| 74 | https://resourcecentre.savethechildren.net/pdf/working_together_-_including_children_in_research_on_violence_against_children.pdf/ |  | Working together: including children in research on violence against children – a resource pack for research practitioners | 7 | 1 | 8 |
| 75 | https://www.studentminds.org.uk/uploads/3/7/8/4/3784584/cpdn_document_artwork.pdf |  | Co-producing mental health strategies with students: a guide for the higher education sector | 4 | 0 | 4 |
| 76 | https://www.savethechildren.org.uk/content/dam/global/reports/education-and-child-protection/so-you-want-to-involve-children-in-research.pdf |  | So you want to involve children in research? A toolkit supporting children’s meaningful and ethical participation in research relating to violence against children | 8 | 9 | 17 |
| 77 | https://www.youthpolicy.org/wp-content/uploads/library/2008_Youth_Participation_Guide_Eng.pdf |  | Youth participation guide: assessment, planning, and implementation | 11 | 8 | 19 |
| 78 | https://www.savethechildren.org.uk/content/dam/global/reports/young-people-as-researchers.pdf |  | Young people as researchers | 1 | 0 | 1 |
| 79 | https://resourcecentre.savethechildren.net/pdf/effective_peer_education.pdf/ |  | Effective peer education: working with children and young people on sexual and reproductive health and HIV-AIDs | 2 | 0 | 2 |
| 80 | https://resourcecentre.savethechildren.net/pdf/me_toolkit_children_leaflet.pdf/ |  | What you should expect from adults when they work with you: a guide for children and young people | 1 | 0 | 1 |
| 81 | https://resourcecentre.savethechildren.net/pdf/me_toolkit_booklet_1.pdf/ |  | A toolkit for monitoring and evaluating children’s participation: introduction. Booklet 1 | 4 | 0 | 4 |
| 82 | https://puskapa.org/assets/uploads/2021/07/Handbook-for-Childrens-Participation-in-Research-1.pdf |  | Handbook for children’s participation in research in Indonesia | 13 | 2 | 15 |
| 83 | https://www.youthpower.org/sites/default/files/YouthPower/files/resources/YOUTH%20ADVISORY%20COUNCIL_8%20STEPS%20final.pdf |  | Youth advisory councils: eight steps to consider before you engage | 1 | 0 | 1 |
| 84 | https://www.ncbi.nlm.nih.gov/pmc/articles/PMC6737761/ |  | Co‐producing research with youth: the NeuroOx young people's advisory group model | 1 | 0 | 1 |
| 85 | https://www.pacer.org/parent/php/php-c245.pdf |  | Youth advisory board toolkit | 1 | 0 | 1 |
| 86 | https://resourcecentre.savethechildren.net/pdf/2645.pdf/ |  | A parrot on your shoulder a guide for people starting to work with orphans and vulnerable children | 1 | 0 | 1 |
| 87 | https://www.healthyteennetwork.org/resources/true-you-maryland/yab-recruitment-kit/ |  | Youth advisory board recruitment kit | 1 | 0 | 1 |
| 88 | https://www.rcpch.ac.uk/sites/default/files/2018-09/recipes_for_engagement_2018.pdf |  | Recipes for engagement | 1 | 0 | 1 |

**Supplementary Table 3**

*Frequencies of Scaled Scores of Domains^[[1]](#footnote-1)^*

| **Domains** | **Scaled scores of domains** | **Frequencies of scaled scores** |
| --- | --- | --- |
| **Domain 1: Scope and purpose** | 0 | 3 (3.4%) |
|  | 16.6 | 4 (4.5%) |
|  | 33.3 | 24 (27.3%) |
|  | 50.0 | 8 (9.1%) |
|  | 66.6 | 26 (29.5%) |
|  | 83.3 | 7 (8.0%) |
|  | 100.0 | 16 (18.2%) |
| **Domain 2: Stakeholder involvement** | 0 | 30 (34.1%) |
|  | 16.6 | 4 (4.5%) |
|  | 33.3 | 18 (20.5%) |
|  | 50.0 | 16 (18.2%) |
|  | 66.6 | 10 (11.4%) |
|  | 83.3 | 6 (6.8%) |
|  | 100.0 | 4 (4.5%) |
| **Domain 3: Rigour of development** | 0.0 | 23 (26.1%) |
|  | 6.2 | 21 (23.9%) |
|  | 12.5 | 17 (19.3%) |
|  | 18.7 | 8 (9.1%) |
|  | 25.0 | 8 (9.1%) |
|  | 31.2 | 5 (5.7%) |
|  | 37.5 | 2 (2.3%) |
|  | 43.7 | 2 (2.3%) |
|  | 50.0 | 1 (1.1%) |
|  | 75.0 | 1 (1.1%) |
| **Domain 4: Clarity of presentation** | 0.0 | 7 (8.0%) |
|  | 33.3 | 39 (44.3%) |
|  | 66.6 | 34 (38.6%) |
|  | 100.0 | 8 (9.1%) |
| **Domain 5: Applicability of recommendations** | 0.0 | 24 (27.3%) |
|  | 12.5 | 22 (25.0%) |
|  | 25.0 | 18 (20.5%) |
|  | 37.5 | 13 (14.8%) |
|  | 50.0 | 5 (5.7%) |
|  | 62.5 | 5 (5.7%) |
|  | 75.0 | 1 (1.1%) |
| **Domain 6: Editorial independence** | 0.0 | 78 (88.6%) |
|  | 25.0 | 1 (1.1%) |
|  | 50.0 | 7 (8.0%) |
|  | 100.0 | 2 (2.3%) |

1. : The 'Scaled Scores of Domains' column indicates the scaled scores for each domain, ranging from 0 to 100, where 0 represents the lowest score and 100 represents the highest score. A scaled score is a standardised score derived from the sum of individual scores given for various items within each domain. These sum scores are then scaled to a percentage of the maximum possible score for that domain. This allows for a standardised comparison of performance across different domains, despite variations in the number of items or the maximum possible score for each domain. The 'Frequencies of Scaled Scores' column shows the number of times each scaled score occurred within each domain across all included guidelines. For example, in the 'Scope and Purpose' domain, the scaled score of 33.3 occurred 24 times, meaning that 24 guidelines were assigned a score of 33.3 for this domain. A scaled score of 33.3 indicates that the guidelines received a score equivalent to 33.3% of the maximum possible score for that domain, suggesting a moderate level of adherence or fulfilment of criteria within that specific domain. [↑](#footnote-ref-1)
